# Degree of Housing Instability Shows Independent “Dose-Response” With Virologic Suppression Rates Among People Living With Human Immunodeficiency Virus

**DOI:** 10.1093/ofid/ofy035

**Published:** 2018-03-14

**Authors:** Angelo Clemenzi-Allen, Elvin Geng, Katerina Christopoulos, Hali Hammer, Susan Buchbinder, Diane Havlir, Monica Gandhi

**Affiliations:** 1 Division of HIV, Infectious Diseases and Global Medicine, University of California, San Francisco; 2 San Francisco Department of Public Health, California

**Keywords:** homelessness, housing status, disparities, virologic suppression

## Abstract

Housing instability negatively impacts outcomes in people living with human immunodeficiency virus (PLHIV), yet the effect of diverse living arrangements has not previously been evaluated. Using 6 dwelling types to measure housing status, we found a strong inverse association between housing instability and viral suppression across a spectrum of unstable housing arrangements.

Housing instability constitutes a major structural barrier to treatment outcomes among people living with human immunodeficiency virus (PLHIV) [1–3], and it severely restricts the benefits of achieving complete virologic suppression for both the individual and for public health in terms of reducing rates of forward transmission [4]. Nationally, and specifically in the West, homelessness and housing instability are on the rise [5], threatening the benefits of antiretroviral therapy for PLHIV [6].

However, of the current studies evaluating the impact of housing status among PLHIV, oversimplification of living arrangements that constitute homelessness or marginal housing introduces imprecision to these evaluations. Most of the research to date has dichotomized housing status into homeless versus not, which may underestimate the nuanced effects of disparate living arrangements (eg, transitional housing, shelters, hotels, “couch surfing,” living outdoors) on outcomes. A more recent evaluation of viral suppression rates among PLHIV in clinics receiving Ryan White Care funding used a 3-level categorization of housing (stable, temporary, unstable) and found that the rate of virologic suppression decreased as housing instability increased [7].

To perform a more granular evaluation of the impact of various states of housing stability on HIV treatment outcomes, we performed a large study examining the association between 6 different categories of living arrangements and virologic suppression rates among PLHIV receiving care in a large San Francisco-based HIV clinic.

## METHODS

In 2017, patients self-reported dwelling type upon appointment check-in at a safety-net HIV clinic providing HIV and primary care in San Francisco (“Ward 86”) by circling current housing status on a pictorial survey (see Supplementary Figure) depicting 6 different living arrangements: (1) Rent/Own; (2) Treatment/Transitional Program; (3) Hotel/Single Room Occupancy (SRO); (4) Staying with Friend (“Couch-Surfing”); (5) Homeless Shelter; (6) Outdoors/In Vehicle. Viral load (VL) measurements performed closest to survey completion ±90 days were abstracted from the medical record. We defined virologic suppression as HIV ribonucleic acid level <200 copies/mL. Patients without VL measures within this window were categorized as nonsuppressed. We tabulated descriptive statistics of baseline demographics. We calculated the proportion achieving virologic suppression and mean VL in each housing status category. Using logistic regression, we then calculated the unadjusted and adjusted odds of virologic suppression by the 6-category variable of housing status, while controlling for age by decade (<30, 30–40, 40–50, >50 years), gender (female/male), and race/ethnicity (Black, White, Latino, or other). Two sensitivity analyses were performed to (1) exclude participants with missing VL data from the sample and (2) categorize participants with missing VLs within 90 days of the housing survey as virologically suppressed instead of nonsuppressed.

## RESULTS

**Figure 1.**
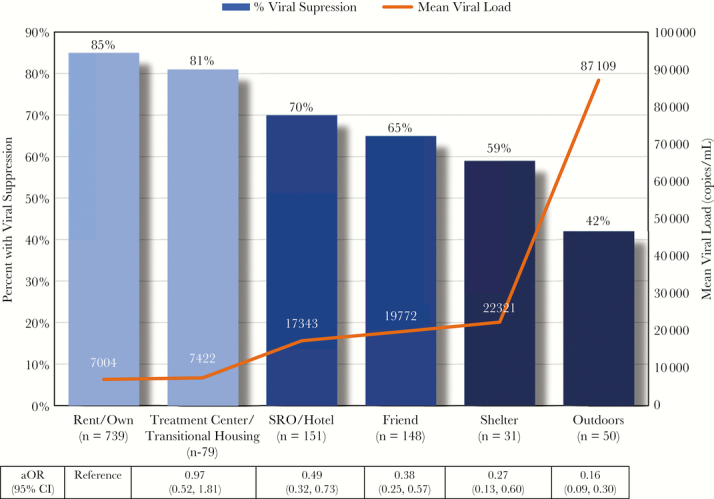
Percent of Patients with Viral Suppression and Mean Viral Load by Living Arrangement among PLHIV at Ward 86 (N=1222). n=total number of patients within each category of housing status. N=total number of patients evaluated. aOR = Adjusted Odds Ratio of Viral Suppression. CI = 95% confidence interval.

From February to July, 2017, 1222 patients at Ward 86 completed the survey, 31 of whom had no VL data within the prespecified window. Median age was 51 years (interquartile range, 41–58): 13% were female, 40% were white, 25% were black, 26% were Latino, and 9% were other. As assessed by the pictorial survey, 62% of patients rented/owned their place of residence, 6% were in rehabilitation, 13% were in an SRO/hotel, 12% were staying with friends, and 7% were staying in a shelter or on the streets. Across a continuum of housing types, virologic suppression rates ranged from 85% (rent/own) to 42% (outdoors), and the mean VL varied from 7004 copies/mL (rent/own) to 87109 copies/mL (outdoors) (Figure 1). Greater housing instability was associated with lower rates of virologic suppression and higher mean VL in a “dose-response” fashion.

In both unadjusted and adjusted models (retaining age, race/ethnicity, gender, see Supplementary Table for models), an increasing degree of unstable housing (eg, SRO/hotel, staying with friends, shelter and outdoors) was associated with a statistically significant lower odds of achieving virologic suppression compared with those who rented/owned (all *P* < .005) (Figure 1). The odds of virologic suppression for those staying in an SRO/hotel was half the odds for those who rented/owned (odds ratio [OR], 0.49; 95% confidence interval [CI], 0.32–0.73); the OR for virologic suppression for those living outside was 0.16 (95% CI, 0.09–0.30). The odds of achieving virologic suppression for those who lived in supportive treatment/transitional housing was not statistically significantly different than in those who rented/owned (*P* = .76). Being African American was associated with lower rates of virologic suppression compared with being white, even when adjusting for housing status (OR, 0.67; 95% CI, 0.46–0.99; *P* = .044). These differences were not seen in those of Latino ethnicity (OR, 0.90; 95% CI, 0.61–1.33; *P* = .17) or “other” race/ethnicity (OR, 0.98; 95% CI, 0.57–1.69; *P* = .20). Age and gender did not demonstrate a statistically significant relationship to virologic suppression in both unadjusted and adjusted models (results not shown). Overall results remain unchanged in both sensitivity analyses.

## DISCUSSION

Our study demonstrates a strong association between dwelling type and virologic suppression rates among PLHIV across a continuum of unstable housing arrangements, a finding that has not previously been described using multiple categorizations of housing. Although living outdoors was associated with the lowest likelihood of virologic suppression, other forms of instability (including living in a shelter, couch-surfing, and being in an SRO) were also associated with lower rates of virologic suppression compared with being stably housed.

Our study is consistent with national and municipal epidemiologic data showing a strong and persistent association of virologic outcomes with housing status, but it provides more granularity on housing type than any prior evaluation [1, 7]. Moreover, our study confirms persistent disparities in virologic outcomes by race, a finding that is independent of housing status in this large urban clinic population. Race disparities in outcomes has been seen in multiple previous studies and is an area of active investigation [8, 9], including by our group. Of note, one limitation of our analysis is that we did not adjust for every factor that could be associated with virologic suppression (eg, adherence) due to lack of availability of such covariate data in this clinic-based cohort and likely significant collinearity with housing status.

## CONCLUSIONS

The National HIV/AIDS Strategy and the HIV/AIDS Bureau of Health Resources Service Administration supports increasing stable housing for PLHIV. Two oft-quoted randomized controlled trials of the impact of housing interventions on virologic outcomes among PLHIV experiencing homelessness demonstrated little improvement by providing permanent “housing alone”, however, likely reflecting the complications of defining housing status and concomitant conditions (both predisposing and causative) not solely addressed by providing nontemporary shelter [10, 11]. As our study suggests, evaluation of the causes of virologic nonsuppression by dwelling type will address a fundamental research gap, which, in turn, should enhance our ability to design interventions to increase virologic suppression across a spectrum of unstable housing arrangements, not just among those living on the street. Incremental improvements in housing stability may improve virologic suppression and reduce transmission rates among PLHIV, and further evaluation of predisposing factors that lead to a variety of unstable housing arrangements is indicated.

## Supplementary Data

Supplementary materials are available at *Open Forum Infectious Diseases* online. Consisting of data provided by the authors to benefit the reader, the posted materials are not copyedited and are the sole responsibility of the authors, so questions or comments should be addressed to the corresponding author.

ofy035_suppl_supplementary_materials
