# Erratum

**DOI:** 10.1093/ofid/ofy271

**Published:** 2018-11-01

In the March 2018 issue of the journal, [Degree of Housing Instability Shows Independent “Dose-Response” With Virologic Suppression Rates Among People Living With Human Immunodeficiency Virus, *Open Forum Infect Dis*, 5(3): ofy035], Figure 1 was missing from this article. This has now been corrected online.

