# Supplementary material for: Degree of Housing Instability Shows Independent “Dose-Response” With Virologic Suppression Rates Among People Living With Human Immunodeficiency Virus
Source: Open Forum Infect Dis. 2018 Mar 14;5(3):ofy035. doi: 10.1093/ofid/ofy035 (PMC5850870; doi:10.1093/ofid/ofy035)
Supplement: ofy035_suppl_supplementary_materials [file ofy035_suppl_supplementary_materials.docx]

**Supplemental Figures/Tables**

Figure: San Francisco Health Department Housing Status Survey


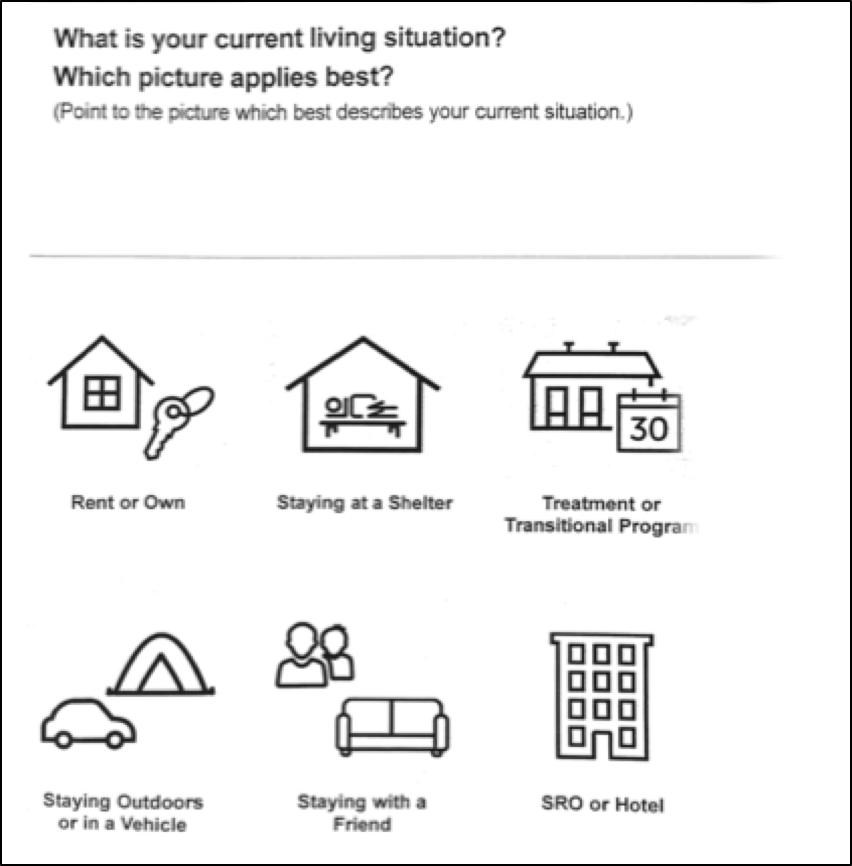


**Table: Odds of Virologic Suppression (Adjusted and Nonadjusted) by Housing Status (N= 1222)**

| Characteristic | Unadjusted OR (95% CI) | p-value | Adjusted OR (95% CI) | p-value |
| --- | --- | --- | --- | --- |
| Housing Status  Rent/Own  Treatment Program  Hotel/SRO  Friends  Shelter  Outdoors | Reference  0.74 (0.40, 1.36)  **0.40 (0.26, 0.60)**  **0.30 (0.20, 0.45)**  **0.25 (0.12, 0.53)**  **0.12 (0.07, 0.23)** | Reference  0.33  **<0.001**  **<0.001**  **<0.001**  **<0.001** | Reference  0.92 (0.48, 1.76)  **0.47 (0.30, 0.72)**  **0.34 (0.22, 0.52)**  **0.26 (0.12, 0.59)**  **0.15 (0.08, 0.29)** | Reference  0.80  **<0.001**  **<0.001**  **<0.001**  **<0.001** |
| Female Gender | 1.36 (0.90, 2.04) | 0.15 | 1.40 (0.87, 2.27) | 0.17 |
| Race/Ethnicity  Black  White  Latino  Other | Reference  1.29 (0.92, 1.82)  1.15 (0.80, 1.66)  1.08 (0.66, 1.79) | Reference  0.14  0.46  0.75 | Reference  **1.48 (1.01, 2.18)**  1.34 (0.88, 2.04)  1.45 (0.82, 2.58) | Reference  **0.044**  0.17  0.20 |
| Age  <30  30-40  50-60  >60 | Reference  0.71 (0.40, 1.26)  0.89 (0.51, 1.56)  1.76 (1.02, 3.03) | Reference  0.24  0.69  0.42 | Reference  0.51 (0.26, 1.02)  0.58 (0.30, 1.14)  0.96 (0.50, 1.86) | Reference  0.058  0.11  0.91 |

OR = odds ratio; CI = confidence interval; SRO= Single Room Occupancy. Bold-face font indicates p-value <0.05.
